# OVOL2 impairs RHO GTPase signaling to restrain mitosis and aggressiveness of Anaplastic Thyroid Cancer

**DOI:** 10.1186/s13046-022-02316-2

**Published:** 2022-03-25

**Authors:** Mila Gugnoni, Gloria Manzotti, Emanuele Vitale, Elisabetta Sauta, Federica Torricelli, Francesca Reggiani, Mariaelena Pistoni, Simonetta Piana, Alessia Ciarrocchi

**Affiliations:** 1 Laboratory of Translational Research, Azienda USL-IRCCS Di Reggio Emilia, Reggio Emilia, Italy; 2 Clinical and Experimental Medicine PhD Program, University of Modena and Reggio Emilia, Modena, Italy; 3 Department of Electrical, Computer and Biomedical Engineering, University of Pavia, Pavia, Italy; 4 Pathology Unit, Department of Oncology and Advanced Technologies, Azienda USL-IRCCS Di Reggio Emilia, Reggio Emilia, Italy

**Keywords:** Anaplastic thyroid cancer, OVOL2, Epithelial-to-Mesenchymal Transition, Mitosis, Cytoskeleton dynamics

## Abstract

**Background:**

Anaplastic Thyroid Cancer (ATC) is an undifferentiated and aggressive tumor that often originates from well-Differentiated Thyroid Carcinoma (DTC) through a trans-differentiation process. Epithelial-to-Mesenchymal Transition (EMT) is recognized as one of the major players of this process. OVOL2 is a transcription factor (TF) that promotes epithelial differentiation and restrains EMT during embryonic development. OVOL2 loss in some types of cancers is linked to aggressiveness and poor prognosis. Here, we aim to clarify the unexplored role of OVOL2 in ATC.

**Methods:**

Gene expression analysis in thyroid cancer patients and cell lines showed that OVOL2 is mainly associated with epithelial features and its expression is deeply impaired in ATC. To assess OVOL2 function, we established an OVOL2-overexpression model in ATC cell lines and evaluated its effects by analyzing gene expression, proliferation, invasion and migration abilities, cell cycle, specific protein localization through immunofluorescence staining. RNA-seq profiling showed that OVOL2 controls a complex network of genes converging on cell cycle and mitosis regulation and Chromatin Immunoprecipitation identified new OVOL2 target genes.

**Results:**

Coherently with its reported function, OVOL2 re-expression restrained EMT and aggressiveness in ATC cells. Unexpectedly, we observed that it caused G2/M block, a consequent reduction in cell proliferation and an increase in cell death. This phenotype was associated to generalized abnormalities in the mitotic spindle structure and cytoskeletal organization. By RNA-seq experiments, we showed that many pathways related to cytoskeleton and migration, cell cycle and mitosis are profoundly affected by OVOL2 expression, in particular the RHO-GTPase pathway resulted as the most interesting. We demonstrated that RHO GTPase pathway is the central hub of OVOL2-mediated program in ATC and that OVOL2 transcriptionally inhibits RhoU and RhoJ. Silencing of RhoU recapitulated the OVOL2-driven phenotype pointing to this protein as a crucial target of OVOL2 in ATC.

**Conclusions:**

Collectively, these data describe the role of OVOL2 in ATC and uncover a novel function of this TF in inhibiting the RHO GTPase pathway interlacing its effects on EMT, cytoskeleton dynamics and mitosis.

**Supplementary Information:**

The online version contains supplementary material available at 10.1186/s13046-022-02316-2.

## Background

Anaplastic thyroid carcinoma (ATC) is a rare and aggressive tumor. Representing less than 5% of thyroid malignancies, ATC accounts for more than half of thyroid cancer related deaths. ATC mortality is over 80% and the mean survival rate is, in general, less than one year [1]. Morphologically, ATC is composed by undifferentiated cells that even if retaining some features indicatives of their epithelial origin have largely acquired a mesenchymal phenotype [2]. The current hypothesis is that ATCs rarely develop as ex-novo lesions, but rather evolve from pre-existing well-Differentiated Thyroid Carcinoma (DTC) through a process of trans-differentiation that likely relies on the Epithelial-to-Mesenchymal Transition (EMT) process [3–5].

EMT is a fundamental morphogenetic process during embryonic development that is often leveraged by tumor cells to acquire motility and invasive abilities [6]. Indeed, EMT is recognized as a major feature of cancer, especially in the recurrent and metastatic settings, being linked with spreading and resistance to therapies [7–9]. For its correct execution, EMT relies on a highly organized gene expression program, governed by a precise network of transcription factors (TFs). Two groups of functionally competing TFs are known to govern EMT, some promoting and other restraining this process. The latest, when activated, promotes the epithelial phenotype, restraining functional cells’ plasticity and motility, therefore executing the reverse trans-differentiation process known as Mesenchymal-to-Epithelial Transition (MET). Despite its relevance in cancer biology, much less is known for TFs able to promote MET, as compared to TFs inducing EMT.

OVOL2 is a MET-promoting TF that works as downstream hub of several signaling pathways, including Wnt, epidermal growth factor (EGF), and bone morphogenetic protein (BMP)/transforming growth factor β (TGF-β) [10–13]. Many studies indicate a fundamental role of OVOL2 in cancer, where it inhibits EMT and metastasis by suppressing ZEB1 expression in breast cancer and nasopharyngeal carcinoma [14, 15], TWIST in lung cancer [16], c-Myc in cutaneous squamous cell carcinoma [17], and Wnt signaling in colorectal cancer [18].

Despite its essential contribution to EMT regulation and the relevance of this process in ATC [19], still no evidence exists of OVOL2 involvement in this type of cancer.

Here we investigated the role of OVOL2 in ATC. We showed that, beside negatively modulating EMT, OVOL2 impairs ATC growth by restraining proficient cell division. Using RNA-Sequencing we showed that OVOL2 inhibits the expression of a core of genes functionally related to cytoskeleton and structural components of cell division. In particular, we demonstrated that OVOL2 modulates the expression of several RHO GTPases and that these proteins likely represent the connecting thread among the multiple effects of OVOL2 on ATC.

## Materials and methods

### Cell cultures

BCPAP, TPC1 and 8505c were obtained from Dr.Santoro, University of Naples, Nthy-ori 3–1 from Dr.Greco, Foundation IRCCS-INT, Milan. 8305c were purchased from Merck KGaA (Darmstadt, Germany). MDA-T41 were purchased from ATCC (Manassas, Virginia, USA). TPC1 and BCPAP were cultured in DMEM while the other cell lines in RPMI at 37 °C/5% CO2 in medium added with 10% fetal bovine serum (Thermo Fisher Scientific, Waltham, MA, USA) and 1% penicillin – streptomycin (Euroclone, Milan, Italy). MDA-T41 medium was added with non-essential amino acids (Thermo Fisher Scientific, Waltham, MA, USA). All cell lines were routinely tested for Mycoplasma contamination using Lonza™ Mycoalert™ Mycoplasma Detection Kit (Thermo Fisher Scientific, Waltham, MA, USA). Authentication by SNP profiling at Multiplexion GmbH (Heidelberg, Germany) was performed in December 2018 for TPC1, BCPAP and 8505c. Authentication by PCR-single-locus-technology at Eurofins Medigenomix Forensik GmbH (Ebersberg, Germany) for Nthy-ori 3–1 was performed in March 2019.

### Plasmids and cell line establishing

Plasmids expressing human OVOL2 HA-tagged were obtained by subcloning OVOL2-HA coding sequence (CDS) from MSCV-OVOL2-HA [20] into non-inducible lentiviral vector pCDH-CMV-MCS-EF1- copGFP (kind gift of Prof. Bruno Calabretta) and the inducible pCW-Cas9 (gift from Eric Lander & David Sabatini (Addgene plasmid # 50,661; http://n2t.net/addgene:50661; RRID: Addgene_50661)) [21] modified to substitute Cas9 CDS with a Multiple Cloning Site. Both vectors (EV) were cut NheI (blunted) and EcoRI and OVOL2-HA CDS was retrieved with XhoI (blunted) and EcoRI. For OVOL2 CRISPR-interference (CRISPRi) knockdown two sgRNAs (sg1: CACCGTCGCGAGTGAGACCACGCCG, sg2: CACCGTTGACACCGTTATGTTGCA) targeting the OVOL2 promoter and a non-targeting control sgRNA (NT: CTGAAAAAGGAAGGAGTTGA) were cloned in the lentiviral vector Plv-hU6-sgRNA hUbC-dCas9-KRAB-T2a-Puro (gift from Charles Gersbac (Addgene plasmid # 71,236; http://n2t.net/addgene:71236; RRID: Addgene_71236)) [22] with Esp3I (Thermo Fisher Scientific, USA).

For lentiviral infections, HEK239T cells were transiently transfected with one of the aforementioned plasmids together with packaging plasmids. Supernatant was collected 48 h later then used for infection protocol (24 h in a single shot). 8505c cells were transduced with pCW-OVOL2-HA and pCW-EV and, 24 h later, selected with 0,5 µg/ml puromycin (Merck KGaA, Darmstadt, Germany) for 3 days. To perform all the experiments, the derived 8505c- cell lines have been seeded and 16 h later, OVOL2-HA expression induced with 1 µg/ml doxycycline (Merck KGaA, Darmstadt, Germany). 8305c cells were transduced with pCDH-CMV-OVOL2-HA-EF1-copGFP or the corresponding EV, 24 h later, cells were sorted (FACS Melody Cell Sorter, BD Biosciences, Franklin Lakes, NJ, USA) for green florescent protein (GFP) expression. Experiments were performed starting from 24 h after infection.

BCPAP and TPC1 cells were transduced with sg1/sg2/NT—dCas9-Krab expressing vectors and selected with 1 μg/ml of Puromycin (Merck KGaA, Darmstadt, Germany) for 3 days. Experiments were performed immediately after selection.

### siRNA transfection

For silencing experiment, 8505c and 8305c cells were reverse transfected with RNAiMax Lipofectamine (Thermo Scientific, Waltham, MA, USA) 24 h prior to seeding for proliferation or wound healing assays. RNA for silencing validation were collected 48 h after transfection. siRNAs used were Silencer Select siRNA specific for RhoU (ID: s33826) and RhoJ (ID: s32982) and control Silencer Select RNAi Negative Control (Thermo Scientific, Waltham, MA, USA) at a final concentration of 50 nM.

### RNA extraction and quantitative real-time PCR

Total RNA was extracted with Maxwell®RSC simplyRNA Cells (Promega, Madison, WI, USA) and retrotranscribed with iScript cDNA kit (Bio-Rad, Hercules, California, USA). Quantitative Real-Time PCR (qRT-PCR) was performed using Sso Fast EvaGreen Super Mix (Bio-Rad, Hercules, California, USA) in a CFX96 Real Time PCR Detection System (Bio-Rad, Hercules, CA, USA). Relative expression of target genes was calculated using the ΔΔCt method by normalizing to the reference gene expression Cyclophillin A (CYPA). See Additional file 2 Table S1 for qRT-PCR primers.

### Western blot and immunofluorescence

For western blot experiments, cells were lysed with PLB (Promega, Madison, WI, USA) supplemented with Protease Inhibitors cocktail (Bimake, Houston, TX, USA). 30-50 µg of total lysate were analyzed by SDS–PAGE using Bio-Rad apparatus (Bio-Rad, Hercules, CA, USA). Immunoblot detection was performed with the appropriate HRP-conjugated secondary antibodies (GE Healthcare, Piscataway, NJ, USA) and Clarity Western ECL substrate (Bio-Rad, Hercules, CA, USA). For immunofluorescence staining, cells were seeded in 4 well Cell Imaging slides (Eppendorf, Hamburg, Germany) then, at appropriate time points, fixed in 4% PFA in PBS for 15 min at room temperature, permeabilized with 0.1% Triton in PBS 2% BSA for 2 min and blocked with 20% FBS in PBS 2% BSA for 1 h. Next, cells were stained with primary antibody for 2 h at room temperature then with appropriate secondary antibody conjugated with AlexaFluor 594 or AlexaFluor 555 (Thermo Fisher Scientific, Waltham, MA, USA). Actin filaments were stained with AlexaFluor 488 or AlexaFluor 594 conjugated Phalloidin (Thermo Fisher Scientific, Waltham, MA, USA) according with manufacturer instructions. See Additional file 2 Table S2 for antibodies used. Images were captured with Nikon Eclipse microscope (Nikon, Chiyoda, Japan) using 20X and 40X magnification and are representative of at least two experiments.

### Flow cytometry and cell cycle analysis

For the assessment of EpCam positivity, cells were harvested, at the indicated time points, with mild trypsinization, washed with IB buffer (1% BSA, 3 mM EDTA in PBS), resuspended in 500 μl of IB and incubated with 10 μl of anti-EpCam antibody (BD Biosciences, Franklin Lakes, NJ, USA) for 1 h at 4 °C. Then, cells were washed twice with IB, resuspended in 250 μl of IB and analyzed. For assessment of Integrin α5-β5 positivity, cells were harvested with mild trypsinization, washed with ice cold 10% FBS in PBS, resuspended in 500 µl of 10% FBS in PBS and incubated with 1 μg of anti- Integrin α5-β5 antibody (Abcam, Cambridge, UK) for 40 min at 4 °C. Washed cells were resuspended in 100 µl of 10% FBS in PBS and incubated with 1:300 goat anti-mouse secondary antibody conjugated with AlexaFluor 555 (Thermo Fisher Scientific, Waltham, MA, USA) at a for 30 min at 4 °C. Finally, washed cells were resuspended in 500 µl of 10% FBS in PBS and analyzed. See Additional file 2 Table S2 for antibodies’ details. For cell cycle analysis, the hypotonic propidium iodide (PI) method [23] was used. All flow cytometry analysis were performed with FACSCanto™ II Cell Analyzer (BD Biosciences, Franklin Lakes, NJ, USA).

### Proliferation, wound healing, invasion, and real-time imaging

For proliferation assays, cells were seeded in 96-well plates (5000 cells/well in 4 wells for condition), and their confluence was evaluated every 12 h for 4 and 5 days. For wound healing experiments, cells were seeded in IncuCyte® ImageLock 96-well plates (24,000 cells/well in 5 wells for condition), scratch wounds were created using the IncuCyte® WoundMaker, following manufacturer’s instructions. Migration was assessed through the evaluation of wound density every 6 h for 3 days. For invasion assays, scratch wounds were obtained as previously described, then 50 µl of ice-cold Matrigel (Corning, Corning, NY, USA) diluted (1:5 for 8305c and 1:2 for 8505, BCPAP and TPC1) and culture media, were poured over and let harden for 30 min. Invasion was assessed through the evaluation of wound density every 6 h for 4 days. For wound healing and invasion experiments, cells were treated for 2 h with Mitomycin-c 10 µg/ml (Merck KGaA, Darmstadt, Germany) to impair proliferation. For real-time imaging, 36 h after OVOL2 induction, images were taken every 5 min for 2 h (for 8505c) or 3 h (for 8305c). Single images were extrapolated from the obtained videos by DaVinci Resolve 16 editing software (Blackmagicdesign) for Fig. 4C.

All the analyses were performed with the Incucyte® Live-Cell Analysis Systems (Model S3; Sartorius AG, Goettingen, Germany). Data were acquired using 10 × and 20X objective lens in phase contrast. Settings used for each type of analysis are reported in Additional file 2 Table S3.

### 2D colony assay

Five hundred 8505c cells were plated in a 10 cm petri dish and allowed to form colonies for 7 days in presence of doxycycline. Ten thousand BCPAP cells were plated in a 10 cm petri dish and allowed to form colonies for 7 days. Then, colonies were fixed with methanol, colored with Crystal Violet (Merck KGaA, Darmstadt, Germany) and counted with ImageJ software [24].

### Chromatin immunoprecipitation assay (ChIP)

After an over-night treatment with doxycycline, 8505c-OVOL2 and 8505c-EV cells were cross-linked with 1% formaldehyde (Merck KGaA, Darmstadt, Germany) then ChIPs were performed with the SimpleChIP® Enzymatic Chromatin IP Kit Magnetic Beads (Cell Signaling Technology, Danvers, MA, USA) following manufacturer’s instructions. Briefely, cells were lysed, and nuclei sonicated with Bioruptor® Pico sonicator (Diagenode, Denville, NJ, USA). Chromatin was precipitated with magnetic beads and the antibody anti-HA tag Additional file 2 Table S2). The immunoprecipitated DNA fragments were quantified by qPCR (primer sequences in Additional file 2 Table S1). For each experiment, 2% of chromatin used for immunoprecipitation was kept as input control. Each qPCR value was normalized over the appropriate input control and reported in graphs as input % (qPCR value/input value × 100).

### Correlation analysis

For the cancer genome atlas (TCGA) analysis we used R TCGA biolinks package to download and analyze RNAseq data (workflow.type ‘‘HTSeq—FPKM’’) from THCA-TCGA project. Correlation analyses were performed using R Corrplot package and applying Spearman method. Genes included in the “Epithelial signature” and in the “Mesenchymal signature” are listed in Additional file 2 Table S4.

### Library preparation and RNA-sequencing

RNA seq libraries were obtained starting from 100 ng of total RNA following Illumina Stranded TotalRNA Prep Ligation with Ribo-zero Plus protocol (Illumina, San Diego, CA). Sequencing was performed using Illumina NEXSeq high-output cartridge (double-stranded, reads length 75 bp-2 × 75 cycles). Sequencing quality was assessed using the FastQC v0.11.8 software (www.bioinformatics.babraham.ac.uk/projects/fastqc/), showing on average a Phred score per base > 34 in each sample. Raw sequences were then aligned to the human reference transcriptome (GRCh38, Gencode release 35) using STAR version 2.7 [25] and gene abundances were estimated with RSEM algorithm (v.1.3.1). Differential expression analysis was performed using DESeq2 R package [26], considering a False Discovery Rate (FDR) of 5% and excluding genes with low read counts. Heatmap representation and unsupervised hierarchical clustering with a complete linkage method were exploited to graphically depict differentially expressed genes (FDR < 0.05). Significant deregulated genes underwent to enrichment analysis, performed on Gene Ontology Biological Processes and Reactome pathways databases via enrichR package [27]. RNAseq down-regulated genes were imported in STRING and filtered for: network type: full network, minimum required interaction score: highest confidence. Disconnected nodes in the network were hidden. StringApp was used to import STRING network into Cytoscape software and obtain protein–protein network visualization [28, 29].

### Statistical analysis

Statistical analysis was performed using GraphPad Prism Software (version 6.01 for Windows, GraphPad Software, San Diego, CA, USA). Statistical significance was determined using the Student’s t-test. Each experiment was replicated two to three times.

## Results

### OVOL2 restrains ATC aggressiveness impairing cells’ mesenchymal features

**Fig. 1:**
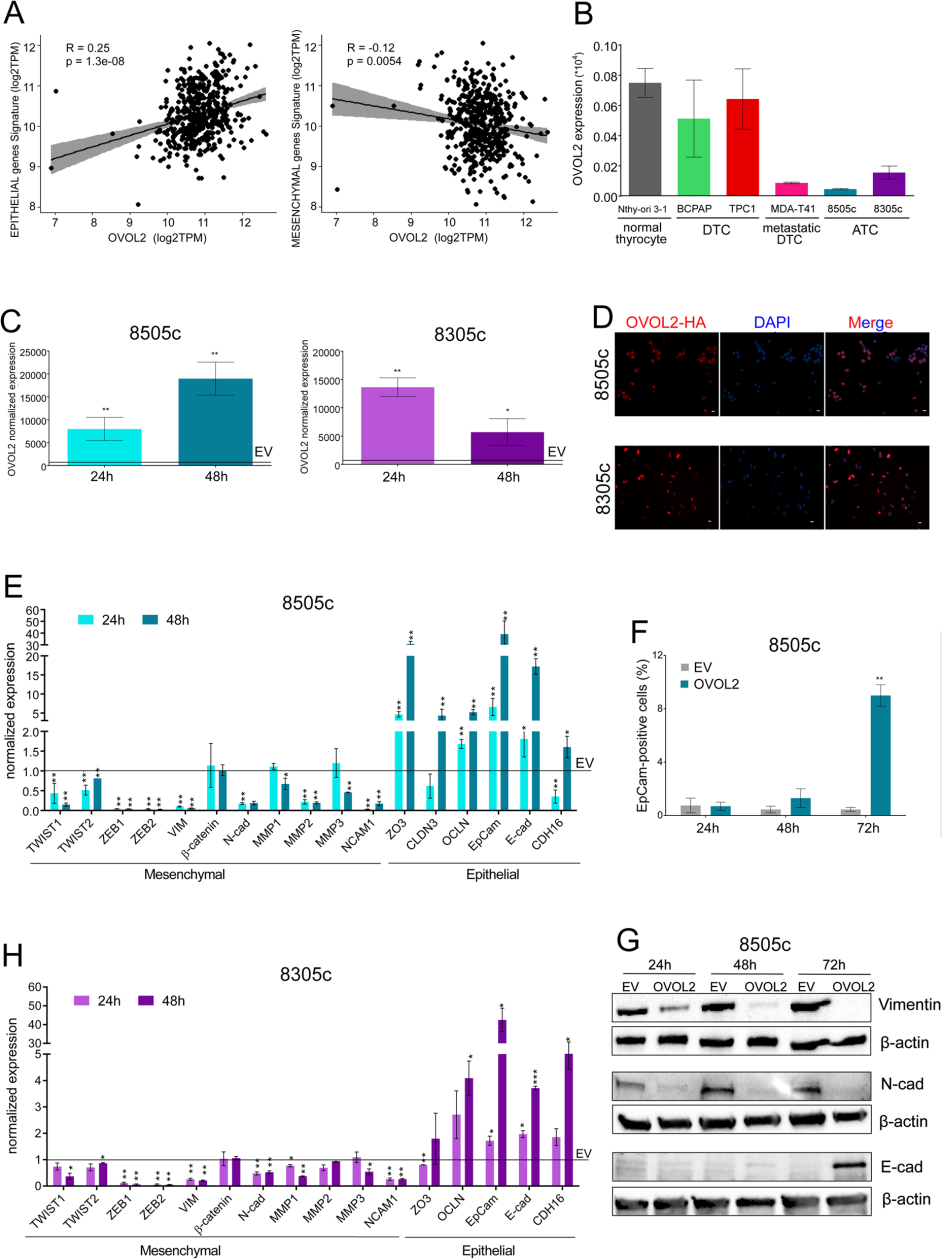
OVOL2 is down-regulated in ATC and its re-expression drives MET transcriptional program. **A** Correlation of OVOL2 expression with Epithelial and Mesenchymal gene signature from the THCA-TCGA database. **B** OVOL2 expression levels in different cell lines derived from immortalized thyrocytes, primary and metastatic Differentiated Thyroid Carcinoma (DTC) and Anaplastic Thyroid Carcinoma (ATC). **C**, **B** Assessment of OVOL2-HA expression by qRT-PCR **C** and IF **D** in 8505c and 8305c cells. IF were performed 48 h after induction. **E**–**G** Evaluation of a panel of epithelial and mesenchymal markers upon OVOL2 induction in ATC cell lines by qRT-PCR (normalized on EV cells) **E**, **H** flow cytometry Staining **F** and western blotting **G**. Histograms represent means and SD of independent biological replicates, *p < 0,05, **p < 0,005

We analyzed the Thyroid Cancer (THCA) gene expression dataset of The Cancer Genome Atlas (TCGA) project to evaluate OVOL2 expression and its potential correlation with EMT features in thyroid cancer. Two short lists gene signatures including the most relevant Epithelial and Mesenchymal markers were defined based on literature [30]. Coherently with its reported role in EMT, OVOL2 showed a significant positive correlation with the expression of epithelial markers (*R* = 0.25, *p* = 1.3e-08), while it was inversely correlated with mesenchymal markers (*R* = -0.12, *p* = 0.0054) (Fig. 1A). This evidence suggests that OVOL2 is significantly expressed only in well-differentiated TC that do not undergo EMT, thus do not have a highly aggressive phenotype. Next, we evaluated OVOL2 expression in TC cell lines corresponding to different histotypes. No significant differences were observed between immortalized normal thyrocytes (Nthy-ori 3–1) and DTC-derived cells (TPC1 and BCPAP). By contrast, in ATC cells (8505c and 8305c) the expression of OVOL2 was barely detectable and metastatic DTC-derived cell line (MDA-T41) showed OVOL2 expression level similar to ATC cells (Fig. 1B).

**Fig. 2:**
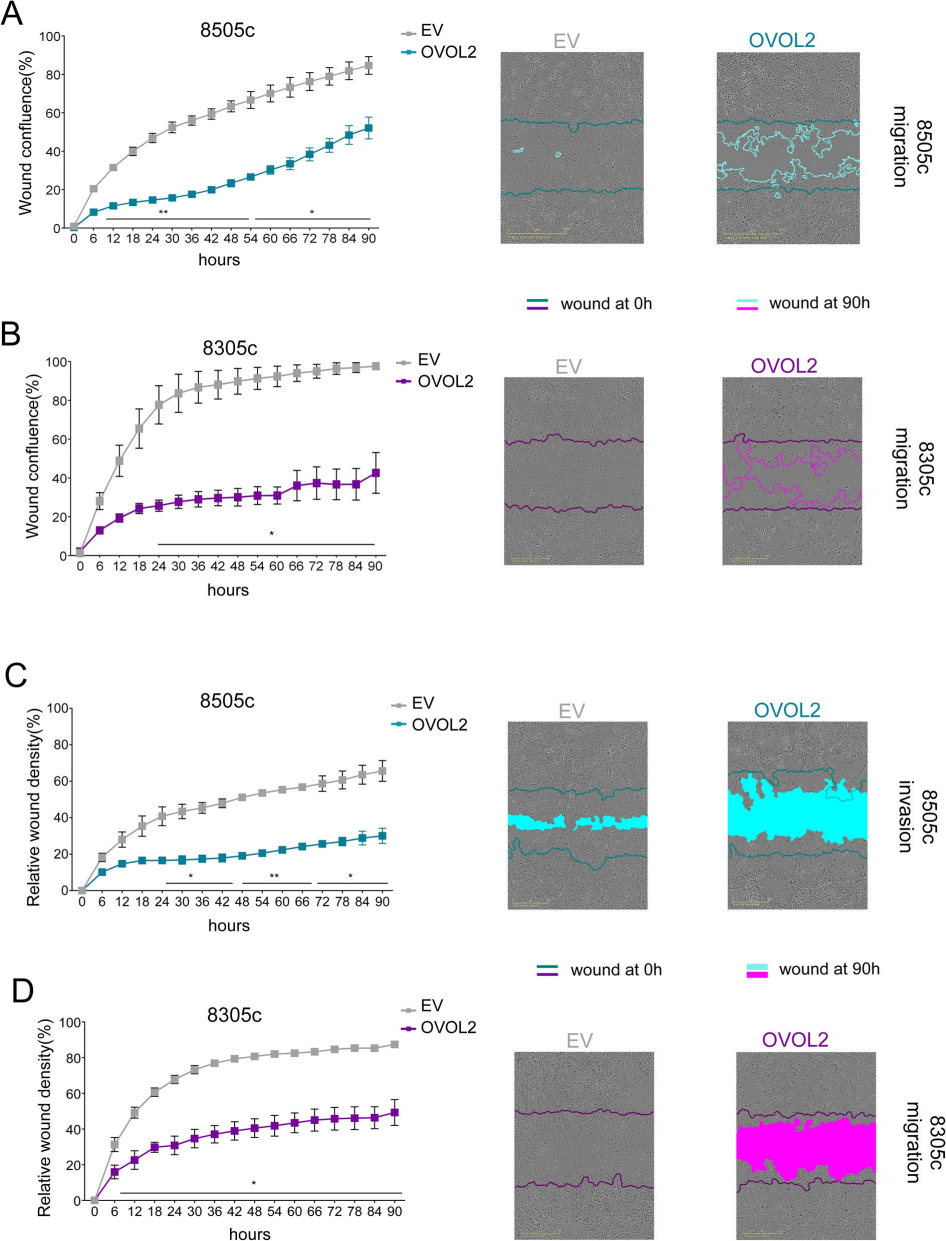
OVOL2 re-expression restrains ATC aggressiveness. **A**-**D**) Effect of OVOL2 re-expression on migration (**A**, **B**) and invasion (**C**, **D**) ability of 8505c and 8305c. Representative images show wound area at the experiment endpoint (90 h). Scale bar 600 µm. Graphics represent means and SD of two independent biological replicates *p < 0,05, **p < 0,005

To better define the role of OVOL2 in restraining aggressiveness, we transduced the ATC-derived cell lines 8505c and 8305c with lentivirus expressing HA-tagged OVOL2, or with the corresponding empty vector (EV) (Fig. 1C-D, Fig. S1 A). We tested the expression of several EMT markers and observed that most mesenchymal markers, including the main mesenchymal cadherin (N-Cad) and its upstream regulators ZEBs and TWISTs were dramatically down-regulated upon OVOL2 expression in both cell lines. Conversely, many surface proteins related to adhesion and epithelial organization were re-expressed as a consequence of OVOL2 induction. Noticeably, re-expression of OVOL2 restored the expression of E-Cad in ATC cells, indicating a profound phenotypic switch toward the epithelial condition (Fig. 1E-H). Wound healing (Fig. 2A-B) and matrix-invasion assays (Fig. 2C-D) were performed to explore whether OVOL2 re-expression affected of ATC cells motility. Indeed, it caused a dramatic reduction (about 50%) of both migration capacity and invasiveness as compared to EV cells, in both cell models.

To consolidate this evidence, we down-regulated OVOL2 expression in DTC cell lines using a CRISPRi approach. Thus, we transduced BCPAP and TPC1 with a lentivirus expressing dCas9-KRAB and two sgRNAs targeting the OVOL2 promoter sequence or with a non-targeting sgRNA (NT) (Fig. S1B). As expected, reduction of OVOL2 expression led to the enhancement of aggressiveness features, including motility and invasion abilities (Fig. S1 C-D).

Together, these data indicate that OVOL2 affects the expression of EMT-related markers and mesenchymal features of TC cells. In particular, these observations suggest that OVOL2 loss could be one of the first steps of the de-differentiation process toward the ATC phenotype.

### OVOL2 affects cell cycle inducing G2/M block by impairing mitosis

**Fig. 3:**
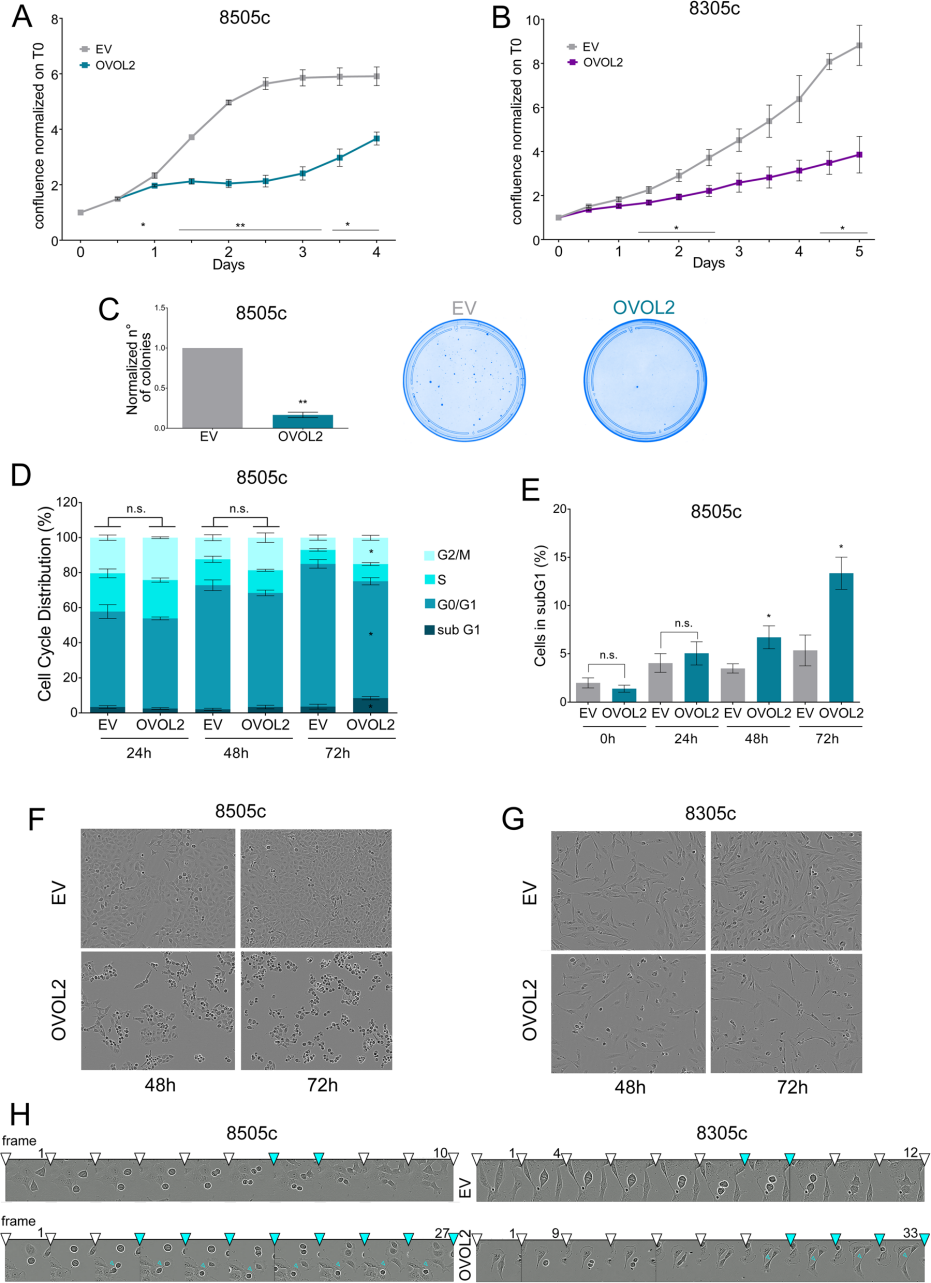
OVOL2 inhibits proliferation, blocks cells in G2/M phase and impairs proper mitosis. **A**, **B** Proliferation assay of EV and OVOL2-expressing 8505c **A** and 8305c **B** cells. **C** Assessment of the ability of 8505c-OVOL2 to form colonies normalized on EV cells. Representative images show colonies at the experiment endpoint, 7 days from the seeding. **D**, **E** Cytofluorimetric analysis of 8505c cells after OVOL2 induction: cell cycle phases distribution **D** and specific % of cells in subG1 upon G0 synchronization **E**. Graphs represent means and SD of independent biological replicates *p < 0,05. **F**, **G** Frames extracted from real-time imaging videos showing altered morphology of 8505c **F** and 8305c **G** 48 and 72 h upon OVOL2 induction compared to EV cells. **H** Frames sequence showing the time requested from EV and OVOL2-induced cells to complete cell division. White arrowheads divide subsequent frames, light blue arrowheads indicate abscission and midbodies. For 8505c EV, 10 consecutive frames are displayed. For 8505c OVOL2 one frame every 3 is displayed, for a total of 27 frames. For 8305c EV, frame 1 and 4 to 12 consecutive frames are reported. For 8305c OVOL2 frame 1 and every 3 frames from 9 to 33 are shown. Consecutive frames are obtained at 5 min from each other

The effect of OVOL2 on ATC cell proliferation was investigated. In 8505c cells, proliferation was rapidly inhibited at 24 h after OVOL2 re-expression (Fig. 3A). In 8305c cells, characterized by a slower doubling time than 8505c, OVOL2 effects on proliferation appeared later but it was consistent with a relevant inhibitory function of this TF on cell growth (Fig. 3B). Also, clonogenicity of 8505c cells was profoundly reduced, OVOL2-cells produced about 80% less colonies compared to EV-cells (Fig. 3C). Conversely, OVOL2 down-regulation enhanced proliferation and colony formation ability of DTC cell lines (Fig. S1 E–F). Cell cycle analysis in 8505c showed that OVOL2 re-expression caused a progressive accumulation of cells in G2/M phase (7% vs 15% at 72 h) and a subsequent increase of the subG1 population compared to EV-cells (3% vs 8% at 72 h) (Fig. 3D), indicating that, failing to complete cell division, OVOL2-cells underwent cell death. To consolidate these observations, we stressed OVOL2 effect on cell cycle by inducing its expression after synchronizing 8505c cells in G0. In this setting, subG1 accumulation of OVOL2-cells occurred earlier and more evident, compared to EV-cells (5% vs 13%) (Fig. 3E and Fig. S1G). Coherently, the expression of G2/M related cyclins and CDK1 was reduced by OVOL2 re-expression, but it was limited and uneven between the two cell lines, suggesting that this was not the direct responsible of the cell cycle block (Fig. S1H).

Morphologically, OVOL2-cells showed a predominant rounded phenotype, barely attaching to the substrate and/or to other cells. This shape is compatible with the rounded mitotic morphology observed when cells are stuck in cell division, after entering M phase (Fig. 3 F, G). Following cell division in real-time (Fig. 3H, Additional file 3), we observed that OVOL2-cells showed major defects in mitosis’ structural features and timing. Compared to EV-cells, they showed a delay in the round-up to initiate M phase, in performing abscission and in re-attaching to substrate after cytokinesis conclusion (EV 50 min vs OVOL2 135 min for 8505c, EV 60 min vs OVOL2 165 min for 8305c). Moreover, chromosomes did not line-up properly on the metaphase plate, mitotic spindle seemed generally instable and poorly organized, leading to cell death after defective division.

**Fig. 4:**
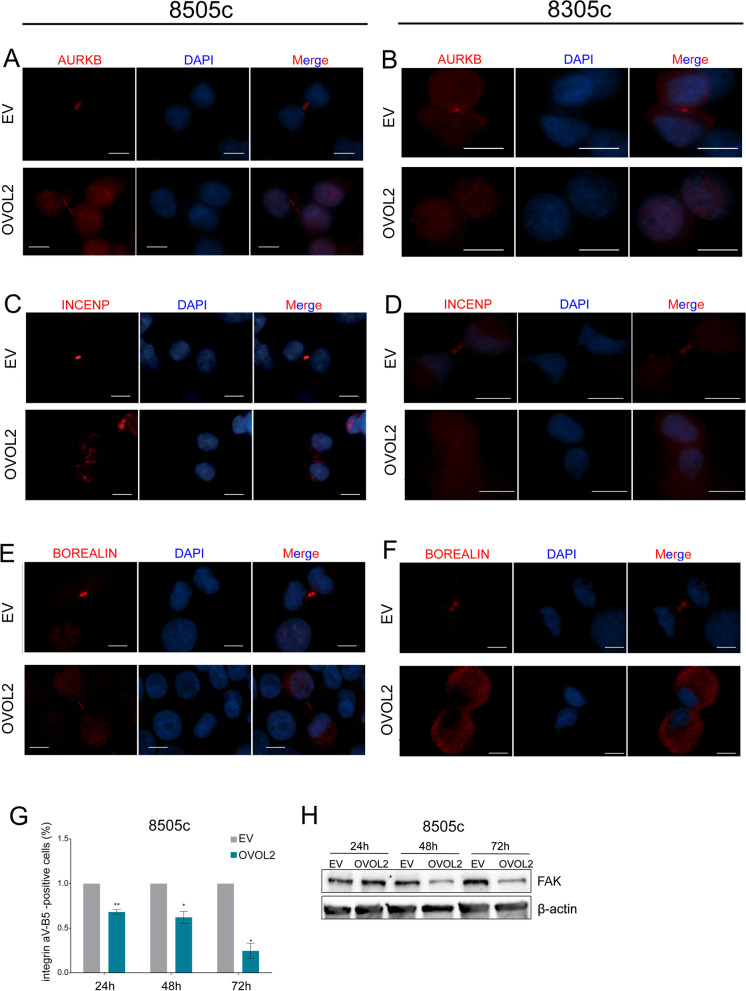
OVOL2 alters mitosis markers localization and extracellular adhesion proteins. **A**-**F** Immunofluorescence staining of AURKB **A**, **B**, INCENP **C**, **D** and BOREALIN **E**, **F** (red) in 8505c and 8305c EV and OVOL2-expressing cells. Nuclei are stained with DAPI (blue). Scale bar 50 µm. **G** Cytofluorimetric analysis for the assessment of integrin αV-β5 positivity in OVOL2-8505c cells normalized on EV cells. Histogram represents mean and SD of two independent biological replicates *p < 0,05, **p < 0,005. H) Western blot analysis of the levels of FAK in 8505c cells at different time points upon OVOL2 induction, compared to EV

Coherently, OVOL2 re-expression produced a massive delocalization of Chromosome Passenger Complex (CPC) components during cytokinesis, in both cell models. In particular, AURKB fails to accumulate on the midbody (Fig. 4 A, B) and INCENP is not localized on the cleavage furrow (Fig. 4 C, D). Notably, also Borealin localization (Fig. 4 E–F) appears diffuse and not correctly gathered. This phenotype is particularly evident in 8305c-OVOL2 cells where it is diffused throughout the cytoplasm with no sign of accumulation at the midbody. Conversely, no evident OVOL2-associated defects on Survivin localization were detected (Fig. S1I).

Reticular Adhesions (RA) and Focal Adhesions (FA) are cytoskeleton structures fundamental for proper cell adhesion during mitosis and interphase, respectively [31–34]. Noticeably, αVβ5 and FAK, major components of the RA and FA respectively, were dramatically decreased in 8505c-OVOL2 cells as compared to EV-cells (Fig. 4 G, H).

Together, these results indicate that OVOL2 alters different crucial steps of G2/M phase, cytoskeleton dynamics organization and cellular adhesion, thus leading ATC cells to death.

### OVOL2-dependent gene program in ATC is linked to mitosis and cytoskeleton organization

**Fig. 5:**
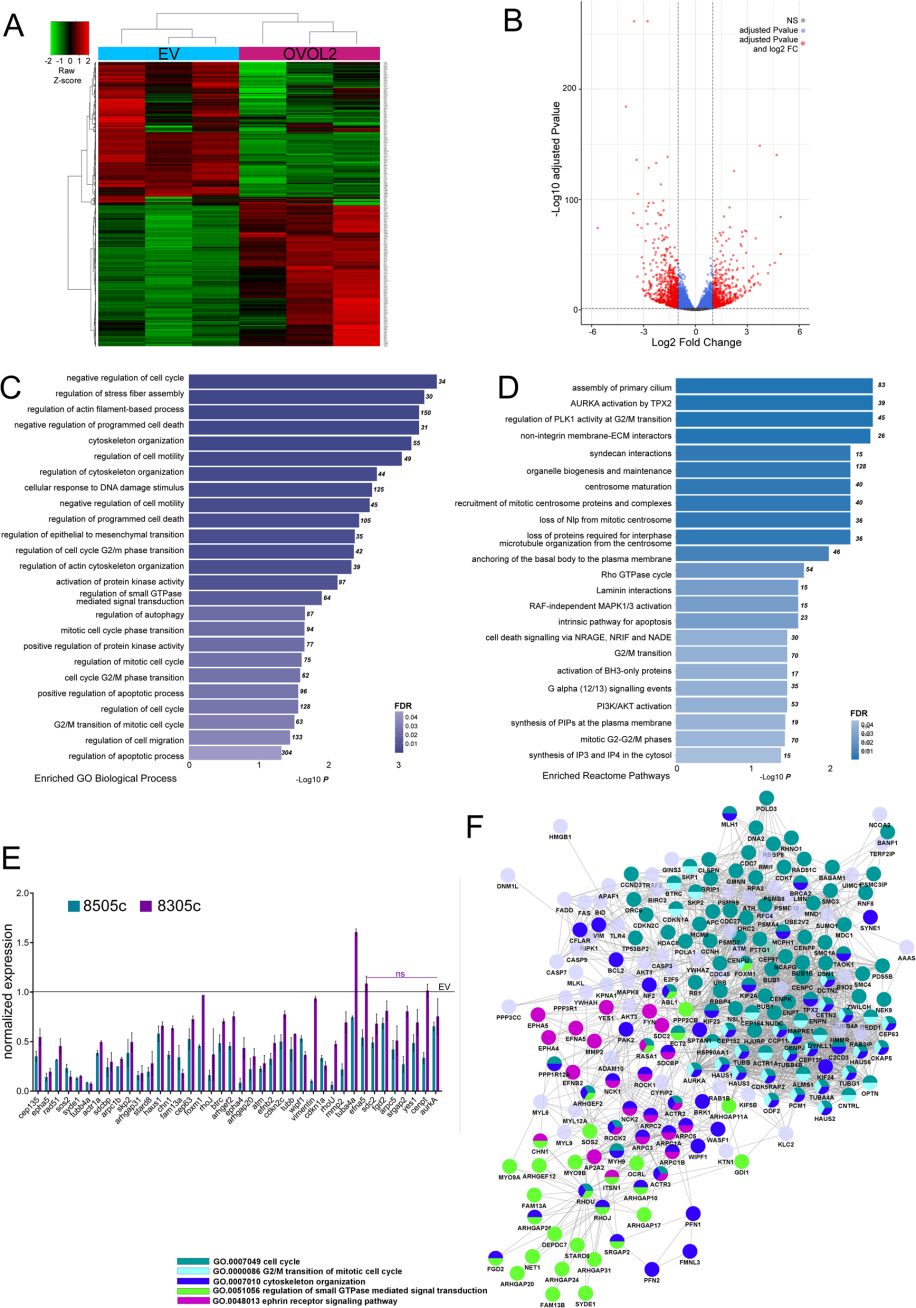
OVOL2 expression impairs pathways related to cell cycle and cytoskeleton dynamics. **A** Heatmap depicting hierarchical clustering based on the 5762 differentially expressed genes, whose read counts are Z-score normalized. Unsupervised hierarchical clustering was performed between 8505c-EV and 8505c-OVOL2 biological replicates. Color intensity for each gene shows Z-score values ranging from red for up-regulation and green for down-regulation. **B** Volcano plot displaying significantly de-regulated genes (adjusted *P* value and log2 FC) between 8505c EV and OVOL2-expressing cells. **C**, **D** GO and Reactome enrichment analysis on OVOL2-deregulated genes from RNAseq analysis. Pathways majorly affected by OVOL2 induction are displayed, ranked for *P* value in each group. Total number of genes enriched in each pathway are reported on the bars. **E** RNA-seq validation of a panel of the most down-regulated genes by qRT-PCR, on 8505c and 8305c 24 h upon OVOL2 induction, normalized on EV cells. All genes are significantly down regulated (*p* < 0,05) unless where the “ns” label is present (*p* > 0,05). Histogram represents means and SD of two independent biological replicates. **F** Protein–protein interaction network of 222 OVOL2 specific targets

To link the observed phenotype to OVOL2 transcriptional function, we performed RNA-sequencing analysis comparing 8505c-OVOL2 to EV cells. Comparative analysis identified 5762 differentially expressed genes (DEGs) of which 2913 were downregulated and 2849 were upregulated, FDR < 0.05 (Fig. 5 A, B). Gene Onthology (GO) and Reactome enrichment analysis revealed that the pathways majorly affected by OVOL2 re-expression were related to migration, motility, cell cycle and mitosis (Fig. 5 C, D). Since OVOL2 is a transcriptional repressor, we focused on the list of downregulated genes. A panel of 40 genes, among the top down-regulated within the most represented GO pathways, were validated in an independent set of experiments, in both 8505c and 8305c cells (Fig. 5E). To construct a network of OVOL2-dependent functionally related genes, down-regulated genes belonging to the pathways most significantly enriched in the RNA-sequencing, were selected for STRING analysis. The resulting 222 OVOL2 target genes were visualized through Cytoscape STRINGapp to define a tight network. Five macrocategories were identified (cell cycle, G2/M transition, cytoskeleton organization, ephrin receptor signaling, regulation of small GTPase mediated signal transduction pathways), coherently with the observed phenotype. Intriguingly, small GTPases emerged among the identified pathways in this analysis. RHO GTPases belong to this family of proteins and are known to be necessary during cytokinesis to orchestrate the correct functioning of cytoskeleton during this phase.

These data suggest that OVOL2-mediated program involves many effectors, part of well-known pathways responsible of proliferation, adhesion, and cytoskeleton dynamics, and that small GTPases signaling could be one of the main elements orchestrating its effects.

### OVOL2-dependent effects are mediated by RhoU transcriptional repression

**Fig. 6:**
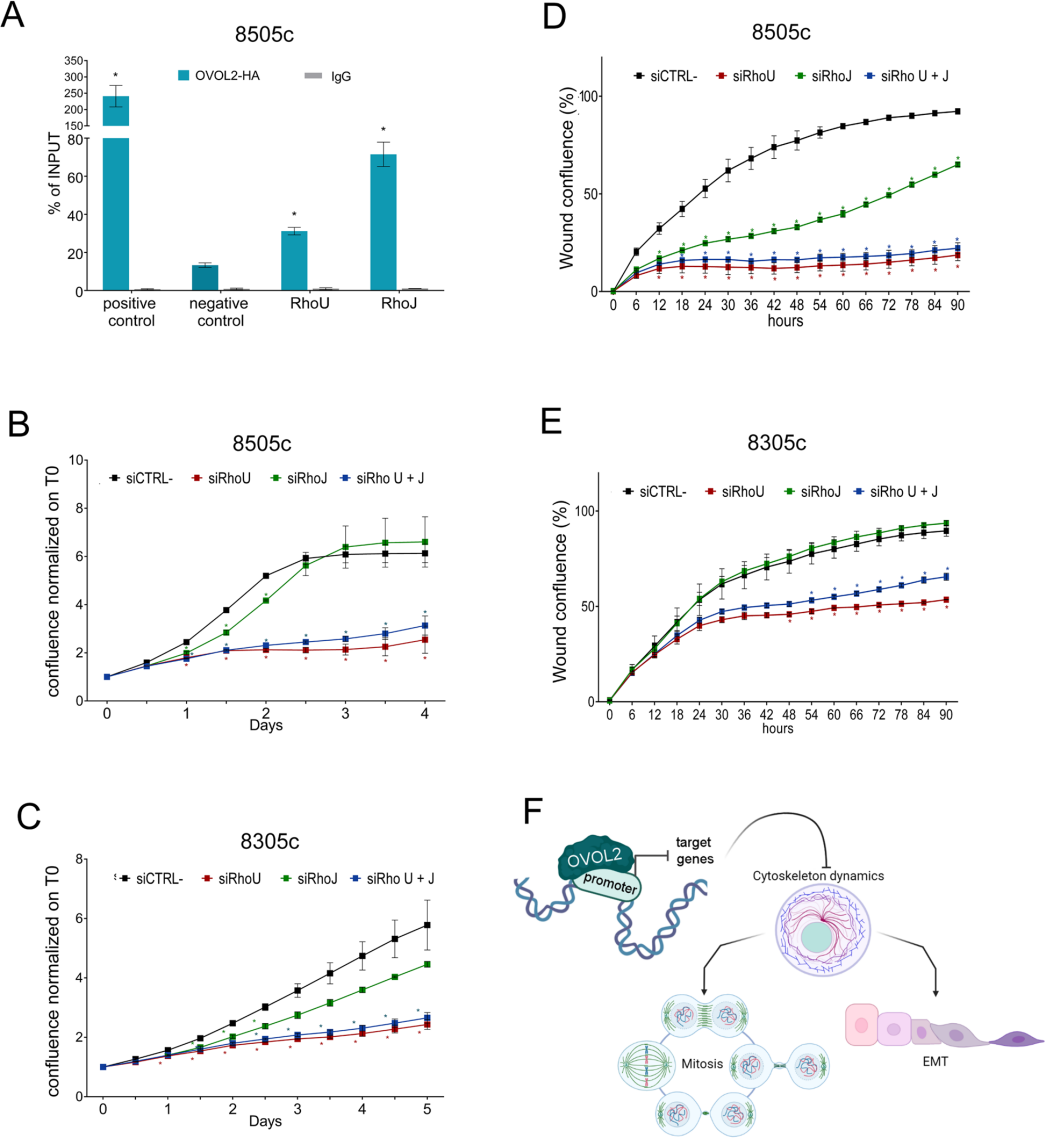
OVOL2 effects depend on RhoU repression. **A** ChIP assay with anti-HA antibody or control normal IgG, in 8505c cells, presented as % of input. An unrelated DNA region was used as negative control while N-cadherin promoter region was used as positive control. Significance is calculated comparing each target to the negative control region. **B**-**E** Proliferation **B**, **C** and migration ability **D**, **E** of 8505c and 8305c respectively, after transfection with siRNA specific for RhoJ, RhoU or the combination of both, compared to control siRNA. Graphs represent means and SD of two independent biological replicates *p < 0,05, **p < 0,005. **F** Schematic representation of the effect of OVOL2 expression on different cellular process in ATC cells (Created with BioRender.com)

RhoU and RhoJ emerged as two major nodes in the OVOL2-dependent functional network. Chromatin ImmunoPrecipitation (ChIP) analysis in 8505c-OVOL2 cells demonstrated that OVOL2 is enriched at the promoter of RhoU and RhoJ (Fig. 6A) proving OVOL2 direct regulation of these genes.

To functional explore the interplay between OVOL2 and these RHO GTPases, we silenced RhoU and RhoJ alone or in combination (Fig. S1J) and analyzed whether the resulting phenotype could mimic the one observed in ATC cell upon OVOL2 re-expression. Silencing of RhoU resulted in a proficient and rapid inhibition of proliferation in both 8505c and 8305c cells (Fig. 6 B, C) that was coherent in extent and timing with the one observed upon OVOL2 induction (Fig. 3 A, B). Conversely, silencing of RhoJ did not significantly affect proliferation of 8505c cells, while had a modest growth inhibitory effect in 8305c cells (Fig. 6 B, C). Simultaneous silencing of RhoU and RhoJ induced an evident cell growth inhibition in both cell models, which was comparable with the one obtained by specific RhoU silencing, further confirming this protein as crucial mediator of OVOL2 function in ATC. Furthermore, silencing of RhoU fully recapitulated OVOL2-dependent inhibition of cell migration in both cell lines (Fig. 6 D, E). In 8505c cells, RhoJ down-regulation led to a significant decrease of cell migration capacity, while its silencing had no effect on 8305c cells (Fig. 6 D, E). Together, these data confirm a direct and functionally relevant link between OVOL2 and these small GTPase and suggest RhoU inhibition as one of the main mediators of OVOL2 effects on ATC cells.

## Discussion

In cancer, OVOL2 loss of function has been reported in many settings and often associated with high aggressiveness and poor patients’ outcome [14, 15, 17, 18, 35–37]. Functionally, this has been linked to its known ability to restrain EMT fostering its reversion through the MET process.

Here, we report a predominant role of OVOL2 in restraining growth and aggressiveness of the deadliest form of TC.

We demonstrated that OVOL2 expression characterizes the well-differentiated forms of TC but is lost in ATC. Re-expression of OVOL2 in ATC cell lines triggered massive changes in the transcriptional program affecting multiple biological functions. In line with its well-known role in cell fate commitment, OVOL2 counteracted EMT inducing re-expression of epithelial-related proteins while restraining mesenchymal features, including cell motility, clonogenicity and invasiveness. Surprisingly, a massive effect was observed on cell proliferation. OVOL2 re-expression reduced cell growth and caused accumulation of cells in G2/M phase with subsequent cell death due to inability of cells to complete division and re-enter cell cycle. Live-cell imaging revealed evident perturbations in the structure and timing of different phases of mitosis. Metaphase and anaphase were affected by improper organization of mitotic spindle which caused delay in chromosome alignment and separation. Moreover, cytokinesis was slowed down and/or not properly completed. This evidence underscored a previously unknown function of OVOL2 in cancer. Indeed, some works reported an association of OVOL2 with inhibition of cancer cells' proliferation, but no mechanistic details or functional validation have been provided so far [37–39].

RNA-sequencing analysis confirmed these data and linked OVOL2 to the direct regulation of a transcriptional program affecting cell cycle, migration, and mitosis. Among the several pathways affected by OVOL2 in ATC cells, we found the small GTPases pathway and RhoU and RhoJ resulted among the top scoring OVOL2 targets in ATC. RHO GTPases are a group of small signaling G-proteins mostly known for their role in regulating intracellular actin dynamics in several biological processes including cytoskeletal dynamics, cell movement and cell division [40–42]. Recently, exome sequencing studies identified cancer-associated alterations in few RHO GTPase family members [43], but activating mutations have low frequency in human cancers [44, 45]. On the contrary, deregulation of their expression is reported in several tumors and correlates with poor prognosis and recurrence [46–52]. Also, several studies demonstrated a prominent role of different RHO GTPases in EMT and metastasis spreading of cancer [53–56]. Moreover, RHO GTPase signaling controls MMPs levels and cell polarity maintenance [57, 58], functions that need to be altered to allow cancer cells to proliferate without control and invade the extra-cellular matrix [59, 60].

Despite this growing attention, transcriptional regulation of these proteins in cancer has only recently been considered as a major aspect contributing to their activity. One of the main TFs associated to RHO GTPases transcription is p53 which strictly connects the GTPases to the DNA damage sensing pathway [61]. Moreover, it is known that RhoU is regulated by Wnt [62], STAT3 [63], RANKL [64], YY1 [65] and Notch [66] while the only TF known to specifically regulate RhoJ is ERG [67].

Here we add another piece of information on the transcriptional control of these proteins demonstrating that OVOL2 represses the expression of RhoU and RhoJ by directing binding on their promoters. We also showed that inhibition of RhoU recapitulates the OVOL2-mediated restraining of cell growth and aggressiveness of ATC cells.

RhoU is a recently discovered RHO GTPase family member. It has a significant sequence homology with Cdc42 but is considered an atypical GTPase because its guanine nucleotide exchange rate is higher than other family members [68]. RhoU plays a role during development by regulating cells’ polarity and architecture [69, 70]. It has been described to be required for maintenance of epithelial integrity, cell adhesion, and maintenance of columnar epithelial morphology [69, 71, 72]. Knockdown of RhoU expression was described to cause abnormal flattening of the mouse foregut epithelium, with loss of the sub-apical cortical actin domain [70]. Arraff and colleagues [73] found that interfering with RhoU activity caused epithelial flattening as well as disruptions in the orientation of coelomic epithelial cells. RhoU has a role also in the FA turnover, in the dismantle of stress fibers and in the formation of filopodia and cell adhesion [70, 74]. Broadly, the RHO GTPase signaling plays a crucial function in the regulation of FA assembly. In turn, FA can activate RHO GTPase cascade through the SRC-FAK signaling [75]. FA are fundamental for the transmission of mechanical forces and regulatory signals between cells and the ECM [76, 77]. Differentiated cells exist in a constant state of tension which maintains tissue architecture and organization [78, 79]. The mechanical tension resulting from basal adhesion and the apical tension is fundamental for proper epithelium organization. FA are composed by different integrins, coupled with FAK proteins, which transmit signals to intracellular cascades. It has been demonstrated its crucial role in modulating integrins and growth factors signaling thus orchestrating cell adhesion and spreading, anchorage-independent growth, apoptosis [80, 81] and preserving epithelial homeostasis during healing/regeneration [82, 83]. Moreover, FA are fundamental in maintaining cell adhesion throughout interphase. Besides, the only remaining adhesion molecules when cells, to perform mitosis, almost completely detach from substrate are RA. They are a specific type of integrin dimers (αVβ5) which have a rapid turnover, are independent from actin filaments [31, 32, 84] and are fundamental for proper division and re-ingression in interphase [85]. Indeed, depletion of RA leads to delayed and defective mitosis and cytokinesis [31]. RA guarantee the maintenance of spatial memory between cell generations, which is fundamental for preserving proper epithelial architecture both during regular cell renewal and in wound healing [86].

Coherently with a significant impairment of FA and RA functions, we observed that in OVOL2 re-expressing ATC cells the overall FAK protein levels were strongly reduced, as well as the levels of the integrins constituting RA.

RhoJ has the specific ability to regulate FA turnover [87] on endothelial cells, identifying its main role in promoting angiogenesis [88]. Its blockage leads to both angiogenesis inhibition and to the disruption of preformed tumor vessels. These cause the functional failure of tumor vascularization [89]. This peculiar characteristic could explain why RhoJ silencing does not impact significantly on proliferation but affect, even if slightly, migration of ATC cells. Its down-regulation, mediated by OVOL2, could mildly affect tumor cells but may have a relevant effect on tumor vascularization, which could not be appreciated by the in vitro approaches we used.

## Conclusions

Taken together, our data demonstrate that OVOL2 re-expression counteracts ATC aggressiveness by acting at multiple levels. OVOL2 effects on cytoskeleton dynamics concurrently impact on two fundamental processes guiding tumor cell biology: EMT and cell cycle regulation (Fig. 6G). The impact of OVOL2 transcriptional repression of RHO GTPase signaling pathway is a brand-new sighting which deserves further investigations to understand its clinical implication.

## Supplementary Information

**Additional file 1: **

**Additional file 2: **

**Additional file 3: **

## Data Availability

The datasets generated and analyzed during the current study are available in the “ArrayExpress” repository, accession number E-MTAB-11055.

## Abbreviations

ATC: Anaplastic Thyroid Cancer
BMP: Bone Morphogenetic Protein
CDS: Coding sequence
ChIP: Chromatin ImmunoPrecipitation
CPC: Chromosome Passenger Complex
CYPA: Cyclophillin A
DEGs: Differentially expressed genes
DTC: Differentiated Thyroid Carcinoma
EGF: Epidermal growth factor
EMT: Epithelial-to-Mesenchymal Transition
EV: Empty Vector
FA: Focal Adhesions
FBS: Fetal Bovine Serum
FDR: Fold Discovery Rate
GFP: Green florescent protein
GO: Gene Onthology
MET: Mesenchymal-to-Epithelial Transition
RA: Reticular Adhesions
TCGA: The Cancer Genome Atlas
TF: Transcription Factor
TGF-β: Transforming Growth Factor β
THCA: Thyroid Cancer
